# Performance Limits of Direct Wideband Coherent 3D Localization in Distributed Massive MIMO Systems

**DOI:** 10.3390/s21103401

**Published:** 2021-05-13

**Authors:** Nenad Vukmirović, Miljko Erić, Petar M. Djurić

**Affiliations:** 1 School of Electrical Engineering, University of Belgrade, 11120 Belgrade, Serbia; nenad.vukmirovic@ic.etf.bg.ac.rs; 2 Innovation Center of the School of Electrical Engineering, University of Belgrade, 11120 Belgrade, Serbia; 3 Vlatacom Institute, 11070 Belgrade, Serbia; 4 Department of Electrical and Computer Engineering, Stony Brook University, New York, NY 11794, USA; petar.djuric@stonybrook.edu

**Keywords:** 5G, wideband direct position estimation, distributed antenna array, Cramér-Rao bounds, mmWave, massive MIMO

## Abstract

We address the accuracy of wideband direct position estimation of a radio transmitter via a distributed antenna array in 5G cellular systems. Our derivations are based only on the presence of spatially coherent line-of-sight (LoS) signal components, which is a realistic assumption in small cells, especially in the mmWave range. The system model considers collocated time and phase synchronized receiving front-ends with antennas distributed in 3D space at known locations and connected to the front-ends via calibrated coaxial cables or analog radio-frequency-over-fiber links. Furthermore, the signal model assumes spherical wavefronts. We derive the Cramér-Rao bounds (CRBs) for two implementations of the system: with (a) known signals and (b) random Gaussian signals. The results show how the bounds depend on the carrier frequency, number of samples used for estimation, and signal-to-noise ratios. They also show that increasing the number of antennas (such as in massive MIMO systems) considerably improves the accuracy and lowers the signal-to-noise threshold for localization even for non-cooperative transmitters. Finally, our derivations show that the square roots of the bounds are two to three orders of magnitude below the carrier wavelength for realistic system parameters.

## 1. Introduction

Recently, there has been a growing interest in massive multiple-input multiple-output (MIMO) systems [1] and millimeter-wave communication (mmWave) technology [2,3,4,5,6,7]. These systems are already finding their place in future 5th generation (5G) cellular systems. A very important application of 5G systems is indoor localization and tracking [8]. Their key objective is to achieve a localization accuracy of about 1cm [9], which is satisfactory for location-based services in cellular systems. In this paper, we aim to obtain the limits of the accuracy of these systems and answer the question of whether, in theory, it is possible to achieve localization accuracy significantly better than the carrier wavelength. The vision is that if such localization accuracy is possible, one could exploit it for location-aided communications. We derived the Cramér–Rao bounds (CRBs) for models that admit only LoS signal components. We addressed two implementations of the system: (a) with deterministic sequences known to the receiver system and (b) with random Gaussian sequences.

The analysis of localization accuracy is important for various reasons [10]. They include location-aided communication (LAC) and coordinated multipoint (CoMP) transmission. For example, CRBs describe local properties for CoMP, and they are much more important than integer carrier wavelength ambiguity. The authors in [11] analyze the performance limits of multiple source localization for known and unknown signals and propose an iterative localization method that breaks the search down into multiple single-source searches. By analyzing the CRB with simulations, one can show that exploiting the coherence between different signal snapshots at the same receiver improves localization accuracy [12]. We explore a different kind of coherence than in [12]—the coherence between signals in different receiving channels in the same signal segment (snapshot). The CRBs for 2D localization of a transmitter that is time-synchronized to the receiving system in a spatially non-coherent scenario with dense multipath propagation is derived in [13].

Few papers have addressed localization in spatially coherent scenarios, and much less the accuracy bounds of such localization. In [14] (RFID—radio frequency identification), Refs. [15,16] (passive source localization), and [17] (MIMO radars—active target localization), the authors have shown that exploiting the carrier phase coupling between all receiving antennas in spatially coherent settings can noticeably increase the localization accuracy. Among these papers, the method in [14] achieves the best precision, which is 3.2% of the carrier wavelength. According to the experimental results in [2], the 60GHz range (mmWave) has great potential for spatially coherent localization, because the non-line-of-sight (NLoS) components are at least 10dB below the LoS component. Methods for passive wideband localization in spatially coherent setups have been proposed in [18], but CRBs for them have not been addressed yet.

In [19], the authors analyze an object inside a reverberation cavity where a wave is reflected a large number of times before arriving at a sensor (NLoS dominant over LoS). An artificial neural network then infers the object’s location from amplitude-only measurements. It was shown that a high dwell time of the wave inside the cavity (the number of reflections) can lower the error even below the carrier wavelength (1/76 of the wavelength). In scenarios that we analyze, we do not rely on a large number of reflections in the area of interest for two reasons—the absorption in the air can have a significant impact on NLoS components in the mmWave band (see [20]) and, besides indoor cells (such as conference rooms, restaurants), we are also interested in small outdoor cells (squares, parks, and so on). Instead, we depend on LoS components (the NLoS components are either negligible or separable from the LoS ones—separable in time thanks to the bandwidth, or separable in space thanks to orthogonal spatial signatures). Note that, even in a small cell/area, the NLoS components with a large number of reflections can have a long propagation distance through the air, which entails that the absorption can be a large source of attenuation for them. As mentioned above, Ref. [2] reported measurements at 60GHz and showed that the NLoS components were at least 10dB below the LoS component. In addition to the mentioned environments (small indoor/outdoor cells/areas), we are interested in the mmWave range of frequencies primarily (especially around 60GHz), where the antennas are small and can be packed more densely, which is suitable for indoor implementations.

In [21], it has been found that the estimation accuracy based on TDoA could significantly be increased by exploiting the carrier phase of arrival. This suggests that the localization accuracy could also considerably benefit from the carrier phases in spatially coherent settings. Algorithms for direct wideband coherent localization of a cooperative transmitter by distributed phased antenna arrays have been reported in [22]. Their performance approaches the CRB, and they solve the ambiguity problem inherent to coherent localization in distributed massive MIMO. There are also direct wideband coherent methods for localization of a non-cooperative transmitter via a distributed antenna array in environments with multipath propagation and interference [18].

In [23], the authors analyzed error bounds for uplink and downlink 3D localization in 5G mmWave systems with an antenna array at the base station, as well as at the user terminal. In the studied scenario, the localization parameters were the directions of departure and arrival. Unlike this setting, ours has a base station array whose antennas are distributed throughout the cell and the localization parameters are the coordinates of a selected user antenna (a user terminal can have more than one antenna).

Numerically less expensive methods for LoS localization in a massive MIMO ultra-dense network are proposed in [24]. They take into account the curvature of the wavefront at the base station arrays (spherical vs. planar). In addition to this curvature, unlike in [24], in this paper, we also consider (1) other useful information due to spatial coherence, and (2) the spatial-wideband effect [25,26]. The localization in our paper is suitable for networks with centralized processing of signals received by distributed antennas such as C-RAN networks [27]. In accordance with [28], we used a physical model for the LoS components and a stochastic model for the NLoS components.

In this paper, we derive the CRBs of localization of an arbitrarily wideband stationary radio frequency (RF) transmitter (Tx) in a spatially coherent scenario (such as the one in [18]). The receiving channels are phase- and time-synchronized, but time synchronization between the Tx and the receiving system is not required, unlike for ToA-based methods [17]. The wavefronts are spherical (the planar wave assumption is not used), contrary to the scenario in [15]. The signals are arbitrarily wideband in the sense that any two TDoAs across the receiving array may differ by more than the reciprocal of the bandwidth. The processing of the received signals is coherent; we exploit the phase differences between all the receiving channels for estimation.

We show that the CRBs are inversely proportional to the squared carrier frequency, and their square roots are two to three orders of magnitude below the carrier wavelength for usual system parameters, as opposed to non-coherent signal scenarios/algorithms, where the ranging/localization CRB is inversely proportional to the squared effective signal bandwidth. Moreover, we show that the CRB is inversely proportional to the square of the signal-to-noise ratio (SNR) for low SNRs when the transmitted sequence is random Gaussian and inversely proportional to the SNR for high SNRs, as well as for known sequences (for any SNR). Finally, we found that the CRB is inversely proportional to the number of samples used for estimation.

The localization system can use an unknown-sequence method (a method suited to random signals) to localize a non-cooperative source in the area of interest, that is completely independent from the system, or a known-sequence method to localize cooperative terminals, especially if they are a part of the communication network that also performs this localization (see different methods in [18]). Known-sequence-based methods can be numerically more complex, but they provide a processing gain which suppresses all interference orthogonal to the searched sequence (similar to CDMA (code-division multiple access) systems). We point out that a system can choose to use unknown-sequence methods in both cases. From the perspective of information theory and attainable accuracy, of interest in this paper; if the localization system knows the sequence, then it has more information at its disposal to localize the source. The latter is reflected in the lower error bounds, as shown in this paper.

This paper follows the research direction of extremely large aperture arrays (a group of antennas distributed in a large area that work coherently), [29], and analyzes them as an enabler for highly accurate 3D positioning. The results presented in this paper are a contribution to the idea of using distributed coherent massive MIMO systems as a component (enabling technology) of future 6G wireless networks [30].

The paper is organized as follows. In the next section, we explain the system and signal model used for deriving the CRBs. In Section 3, we present the main derivation results of the paper, and in Section 4, we provide some numerical results obtained from the derived CRBs. In the last section, we provide concluding remarks and directions for future research. In Appendix A, we explain in detail the derivation of the CRBs when the receiving system knows which sequence/waveform the transmitter uses, whereas in Appendix B, we present the derivation details for random transmitted signals.

In the paper, we use the notation given in Table 1.

Note that, for the most part of this paper, we use normalized time and frequency quantities (such as $f_{c}$), whereas we use quantities in physical units (non-normalized, such as $\nu_{c}$) only when necessary. The reason for this decision is that the transition between the analog and the digital domain is then trivial (s(n)↔s(t), when t=0,1,2,⋯).

## 2. System Model

### 2.1. The Setup

**Figure 1:**
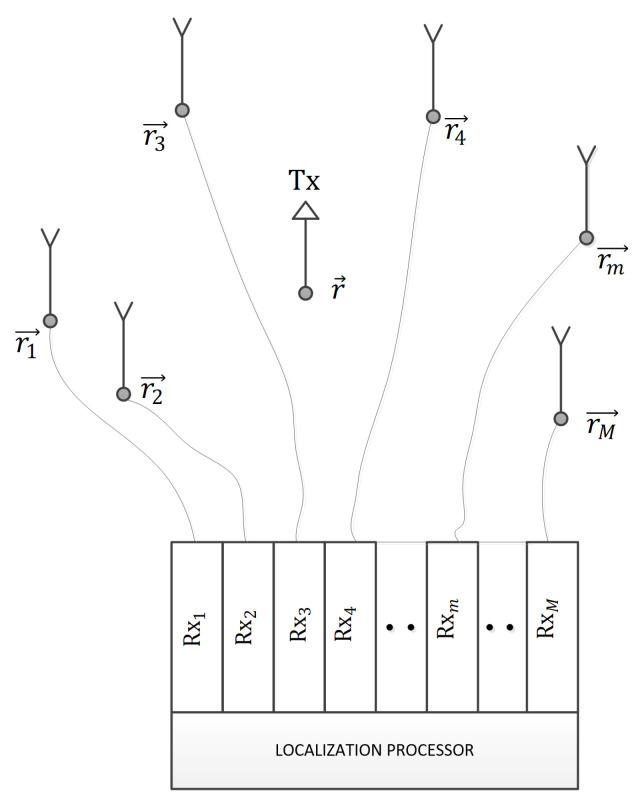
The system model.

A single stationary transmitter, Tx, is at an unknown location r=(x,y,z). A distributed stationary receiving antenna array has *M* elements/channels, ${\mathrm{Rx}}_{m}$, $m\in \left\{1,2\cdots M\right\}$, where the ${\mathrm{Rx}}_{m}$th antenna is at a known location $r_{m}=\left(x_{m},y_{m},z_{m}\right)$, as in Figure 1.

When the distances between adjacent antennas in an array are of the order of $\lambda_{c}/2$, where $\lambda_{c}$ is the carrier wavelength, we call the array a collocated or classical array. If some of the antennas are separated by many wavelengths, we call such an array distributed. Note that a distributed array does not have to contain only independent (single) antennas, but it may also contain collocated arrays (as its subarrays). It may be cheaper to build a distributed array from prefabricated subarrays (with densely packed, i.e., collocated, antennas in each one) and to multiplex the signals from the antennas of the same subarray through a single cable. (This might especially be convenient at higher frequencies.)

Next, we define the spatial coherence of a signal component (such as an LoS component) from a transmitter Tx, wherever it may be in a given area of interest, A, across a set of receiving (Rx) antennas, M. We say that the spatial coherence exists, if the difference, denoted by Δd, in the distances from Rx antennas *m* and *ℓ* to the Tx implies that the difference in the phase of that component at these two Rx antennas is equal to $-2\pi \nu_{c}\Delta d/\tilde{c}$, for any m,ℓ∈M, where $\nu_{c}$ is the carrier frequency and $\tilde{c}$ is the speed of propagation in that medium. Note that this is a weaker condition than the medium being homogenous and isotropic. It is weaker because only the phase differences are required to follow this law and only for signals coming from inside of A. Furthermore, note that spatial coherence is more likely to exist in smaller cells, and especially indoors. Most importantly, note that spatial coherence is a property of the propagation medium only—it has nothing to do with the front-ends or the cables of the Rx system.

We also define coherent localization methods. We call them coherent if they exploit the information in phase differences between signals in each pair of the receiving channels to increase the accuracy of the localization. This requires (a) spatial coherence in the medium and (b) that the Rx channels work coherently (i.e., that they are time-, frequency-, and phase-synchronized). If they do not work coherently, the phases of the received signals may well be scrambled and this information is lost. Non-coherent methods would still work, because they rely only on signal envelopes. There are also semi-coherent methods, which exploit the phase differences between the signals from the same subarray, but not between different subarrays (because, e.g., the subarrays may be far apart from each other, that is, spread out over a large geographical area where spatial coherence does not exist). Note that spatial coherence, in most cases, exists across collocated arrays.

We now define direct localization methods. These methods estimate the location directly from the raw acquired data (the signal samples), whereas indirect methods estimate the location from some intermediate estimates (such as directions of arrival, times of arrival, time differences of arrival, or received signal strengths). Direct methods usually have better performance at the cost of higher numerical complexity and the need for the transference of raw samples to the signal processor. Furthermore, a wideband localization method is able to cope with signals whose bandwidth is large, such that the differences between envelope delays at different Rx antennas may exceed the inverse of the bandwidth.

Next, we draw some comparisons of our model with other models. Our receiving system model is equivalent to the one in [31,32] for a cell-free network, but the propagation model, however, is not. Namely, the network contains antennas, placed over a wide area, which (thanks to synchronization) work coherently to receive signals. Then, the signal processors process these raw signals, but, unlike in [31,32], where the phases in the Rx signals are scrambled, we consider scenarios with spatial coherence of LoS components, which means the information about the Tx location contained in the phases is preserved. The network may have many more antennas, but only a subset of *M* of them is chosen. It is assumed that the Tx is in LoS conditions with low path loss with each of the *M* antennas and that there is spatial coherence of the LoS components across this subset (as explained in [18]). Furthermore, this assumption is made for scalability reasons (see, e.g., [33]). The spatial coherence condition allows coherent localization, which in turn, as we will show, can provide a much higher accuracy. The network also chooses a central processing unit that will run a direct localization algorithm on the acquired signals.

A similar comparison can be drawn against the model in [34]. Using the terminology from [34], we have a number of antennas in a distributed antenna system spread over the area of interest, grouped into remote antenna units (RAUs), where the RAUs can have different numbers of antennas; each RAU contains one or more antennas. The signals from the antennas are fed to baseband processing units (BPUs), as separate streams, where processing occurs (centralized processing is enabled, i.e., a direct localization algorithm can be run). The main differences are (a) the elements of the channel matrix in [34] are random, whereas we model the LoS components separately (from NLoS) and deterministically, and (b) the symbols transmitted at the same time arrive at the Rx antennas also at the same time (narrowband approximation) [34], whereas we consider wideband signals.

We also describe a contrast with [35], where a large number of low-cost low-power sensors transmit wirelessly short messages to a fusion center. Instead of performing coherent direct localization (which requires raw signals at the fusion center, and, therefore, probably requires wired infrastructure), it can cooperate with a high performance network, which performs coherent localization and advanced wireless communication (a network which we model) to help it detect the presence of mobile transmitters.

For the receiving channels to work coherently, the cables connecting the Rx antennas to their front-ends (Figure 1), together with the front-ends themselves, should either induce equal delays (including phase delays) in the signals or the deviations should be compensated for in preprocessing (this is a more likely and a more flexible solution). The IQ (in-phase-quadrature-phase) demodulators should use a local carrier from a common source (frequency-synchronization), the A/D converters should have a common clock, and the corresponding samples should have the same indices (timestamps) (time-synchronization). Readers interested in practical implementations of synchronization methods are referred to [36]. The Rx system receives a signal segment of *N* complex samples (*N* in-phase samples as real plus *N* samples from the quadrature-phase branch as imaginary) from each channel (starting, of course, from the same sample index in each channel). The signal processor then runs a localization algorithm on that segment (which contains MN samples). The SNRs are defined for each channel, and they are expected to decrease with the square of the distance from the Tx to an Rx antennas, but we allow them to have any values (deviations could happen because the polarizations are not aligned perfectly, for example; especially if the antennas are distributed in 3D). The system, of course, chooses a carrier frequency and a bandwidth appropriate for the band it wants to monitor (to perform localization in).

Note that the Tx does not need to be either time- or phase-synchronized with the receiving system. This means that even noncooperative transmitters can be localized. If one hopes to estimate the Tx location with accuracy better than the carrier wavelength, then the positions $r_{m}$ of the receiving antennas have to be determined with even better accuracy before the localization algorithm is executed.

### 2.2. Signal Model

The Tx transmits an RF signal (approximately) bandlimited to $\left(\nu_{c}-B/2,\nu_{c}+B/2\right)$, where $\nu_{c}$ is the carrier frequency in Hz and *B* is the signal bandwidth in Hz. The signal received at the ${\mathrm{Rx}}_{m}$th antenna at a distance $d_{m}$ from Tx, bandlimited to $\left(-B/2,B/2\right)$, is sampled at the Nyquist rate $\nu_{s}=B$ and is represented in a complex form as

(1) $$\begin{array}{cc} {\bar{u}}_{m}\left(n\right) & =a_{m}s_{m}\left(n\right)+{\bar{u}}_{m}^{\mathrm{NLoS}}\left(n\right)+{\bar{w}}_{m}\left(n\right),\\ s_{m}\left(n\right) & =\exp\left(-j\omega_{c}\left(\tau_{0}+\tau_{m}\right)\right)s\left(n-\tau_{0}-\tau_{m}\right),\\ {\bar{u}}_{m}^{\mathrm{NLoS}}\left(n\right) & =\sum_{\ell =1}^{L_{m}}a_{m,\ell }s\left(n-\tau_{0}-\tau_{m,\ell }\right), \end{array}$$

where $n\in \left\{0,1\cdots N-1\right\}$; $a_{m}$ is a real-valued attenuation factor for the LoS component, $s_{m}\left(n\right)$; s(·) is the sequence/waveform; the term ${\bar{u}}_{m}^{\mathrm{NLoS}}\left(n\right)$ models all NLoS components; $\omega_{c}=2\pi f_{c}$ and $f_{c}=\nu_{c}/B$ are the normalized angular and natural carrier frequencies, respectively; the unknown value $\tau_{0}$ models the asynchronism between the Rx system and the Tx (we use, as a reference, the Rx time axis); $\tau_{m}=d_{m}/c$ is the propagation time from Tx to ${\mathrm{Rx}}_{m}$, where $d_{m}=∥r-r_{m}∥$ and ∥·∥ is the Euclidean norm; the normalized propagation speed is $c=\tilde{c}/\nu_{s}$, where $\tilde{c}=3\times 10^{8}\;m/s$; there are $L_{m}$ NLoS paths to the ${\mathrm{Rx}}_{m}$th antenna with complex-valued coefficients $a_{m,\ell }$ and propagation times $\tau_{m,\ell }$; ${\bar{w}}_{m}\left(n\right)$ represents noise; the acquired samples are ${\bar{u}}_{m}\left(n\right)$.

Note that $\tau_{0}$ and $\tau_{m}$ are fractional dimensionless values (the propagation time delays do not have to be integer multiples of the sampling interval) and that the sequence/waveform s(·) is defined on the entire (continuous) range R because it has been transformed into a continuous waveform inside the Tx.

Unlike the signal model in [1,37], the signal model in this paper is spatially wideband. Furthermore, the steering vectors are modeled implicitly by $\tau_{m}$ for each individual Rx antenna. Besides the spatial-wideband effect, this also models the curvature of the wavefronts, which cannot be neglected due to the size of the subarrays compared to the area of interest. Furthermore, in contrast to [1,37], the coefficients $a_{m}$ are real-valued, thanks to the spatial coherence of the LoS components. Note that the coefficients $a_{m,\ell }$ for the NLoS components may have any phase. In [25,26], it is argued that modeling the spatial-wideband effect is important even for collocated massive arrays and that it is critical for distributed systems.

We scale the signal ${\bar{u}}_{m}\left(n\right)$, in a preprocessing step, by $1/a_{m}$, because the signal-to-noise ratios (SNRs) and noise powers are considered to be known to the receiver. Thereby, the useful signals in all the receiving channels have the same powers. Then, we have

(2) $$u_{m}\left(n\right)=s_{m}\left(n\right)+u_{m}^{\mathrm{NLoS}}\left(n\right)+w_{m}\left(n\right),$$

where $u_{m}^{\mathrm{NLoS}}\left(n\right)$ are the (scaled) NLoS components and $w_{m}\left(n\right)$ is a circular-complex zero-mean Gaussian random process with variance $\sigma_{m}^{2}$, $\mathrm{CN}(0,\sigma_{m}^{2})$, independent across time samples and channels and independent from the waveform s(n). Then, the SNR in channel *m* is defined by

(3) $${\mathrm{SNR}}_{m}=\frac{1}{N\sigma_{m}^{2}}\sum_{n=0}^{N-1}{\left|s(n)\right|}^{2}.$$

The propagation attenuations are modeled by $\sigma_{m}^{2}$, but the model considers that no information about the transmitter location is contained in the attenuations. In practice, location estimation based on phases is more robust than the location estimation based on attenuations.

Our objective is to find the CRBs of the estimates of unknown parameters for the cases of (a) known and (b) random sequences. In the former case, the unknown parameters are $\alpha =\;\left(\tau_{0},x,y,z\right)$ and in the latter, $\alpha =\;\left(x,y,z\right)$. Let us recall that the Tx is not time-synchronized with the Rx system. Therefore, the parameter $\tau_{0}$ (the shift between the Tx and Rx time axes) is unknown to the signal processor. Even though we are not interested (in this context) in obtaining an estimate of $\tau_{0}$ (we are only interested in estimating $\left(x,y,z\right)$—the Tx location), $\tau_{0}$ must be modeled as an unknown and, therefore, as a part of α in case (a). Otherwise, an estimation algorithm could exploit this information to produce a better estimate than could be possible (on average) with the given model (and that would mean that the error bound would be different, too).

In contexts outside of the scope of this paper, an estimate of $\tau_{0}$ might not be irrelevant. On the contrary, if the Rx system (such as a base station or a group of them) were synchronized to the universal time, and it sent its estimate of $\tau_{0}$ to the Tx (the terminal), the Tx could adjust its clock to match the universal time as well (as an additional service, the system could provide to the terminal).

Another important thing to note is that, in case (b), regardless of how good or bad an estimation algorithm is, it would be unable to estimate $\tau_{0}$ at all. An intuitive explanation could be: If ${\hat{\tau }}_{0}^{\prime }$, ${\hat{s}}^{\prime }\left(t\right)$, and ${\hat{r}}^{\prime }$ were likely estimates of $\tau_{0}$, s(t), and the location, then the estimates ${\hat{\tau }}_{0}^{\prime \prime }={\hat{\tau }}_{0}^{\prime }-\Delta \tau$, ${\hat{s}}^{\prime \prime }\left(t\right)={\hat{s}}^{\prime }\left(t-\Delta \tau \right)$, and ${\hat{r}}^{\prime }$ would be equally likely, for any Δτ, no mater how large $\left|\Delta \tau \right|$ is, so the error would be unbounded. A formal explanation is as follows: the Fisher information matrix (see the next section) in case (b), if we chose $\alpha =\;\left(\tau_{0},x,y,z\right)$ would be singular, and we need to invert it to obtain the CRB, which would mean that the CRB would tend to infinity.

## 3. Cramér-Rao Bounds

The authors in [23,38] provide CRBs for localization when the user terminal and the base station each have a single collocated (small aperture) antenna array. They show that, when the number of antennas grows, the CRBs of the LoS component parameters tend to the appropriate LoS-only CRBs, because the LoS components become separable from the NLoS ones, thanks to the channel sparsity in mmWave. In accordance with that, we derive the CRBs when $u_{m}^{\mathrm{NLoS}}\left(n\right)=0$. This allows us to quantify the impact of the location information in the LoS components on the attainable localization accuracy, regardless of the model for the NLoS components (whether they are modeled as random or deterministic and whether they are changing more slowly of quickly). Even if the NLoS components are not negligible in some scenarios in practice, the LoS-only CRBs are useful for selecting the resolution of a search grid for localization methods that use grids. If the system designer cannot predict how much the NLoS components would degrade the accuracy, it seems logical to use the resolution appropriate for the case without that degradation.

### 3.1. Known Sequences (Cooperative Transmitter)

This case corresponds to a scenario where a base station allocates a sequence for a cooperative user terminal. The terminal then transmits the sequence so that the base station can localize the terminal. From the assumptions about $w_{m}\left(n\right)$ in (2), we deduce that Rewm(n) and Imwm(n) have zero-mean Gaussian distributions with variance $\sigma_{m}^{2}/2$, $N\left(0,\sigma_{m}^{2}/2\right)$, where Re and Im denote the real and imaginary parts of the argument, respectively. It holds then, that $E\left(u_{m}\left(n\right)\right)=s_{m}\left(n\right)$, where E stands for the expectation operator. Furthermore, the probability density function (PDF) of a single time sample is

(4) $$p\left(u_{m}\left(n\right);\alpha \right)=\frac{1}{\pi \sigma_{m}^{2}}\;\exp\left(-\frac{1}{\sigma_{m}^{2}}{\left|u_{m}\left(n\right)-s_{m}\left(n\right)\right|}^{2}\right).$$

All the observations are given by the vector $u=[u_{1}\left(0\right),\;u_{1}\left(1\right),\cdots ,u_{1}\left(N-1\right),$

$u_{2}\left(0\right),\cdots ,u_{M}\left(0\right),$$u_{M}\left(1\right),\cdots ,u_{M}{\left(N-1\right)]}^{⊤}$. The PDF of u is given by

(5) $$p\left(u;\alpha \right)=\prod_{m=1}^{M}\prod_{n=0}^{N-1}p\left(u_{m}\left(n\right);\alpha \right),$$

and thus, the loglikelihood function, $L=\ln p\left(u;\alpha \right)$, is

(6) $$\begin{array}{c} L=-N\sum_{m=1}^{M}\ln\left(\pi \sigma_{m}^{2}\right)-\sum_{m=1}^{M}\sum_{n=0}^{N-1}\frac{1}{\sigma_{m}^{2}}{\left|s_{m}\left(n\right)-u_{m}\left(n\right)\right|}^{2}. \end{array}$$

The Fisher information matrix (FIM) of the unknown parameters is defined by

(7) $$I\left(\alpha \right)={\left[I_{ij}\right]}_{4\times 4},\;I_{ij}=-E\left(\frac{\partial^{2}L}{\partial \alpha_{i}\partial \alpha_{j}}\right)=I_{ji}.$$

After taking the first partial derivatives of L with respect to the unknown parameters given in α, we get expressions of the form

(8) ∂L∂x=Re∑m=1M∑n=0N−12σm2sm(n)−um(n)*x−xmcdmjωcsm(n)+spm(n),

where (if we use the symbol ${\left(\cdot \right)}^{\prime }$ to denote the first derivative)

(9) $$s_{pm}\left(n\right)=s^{\prime }\left(n-\tau_{0}-\frac{d_{m}}{c}\right)\times \exp\left(-j\omega_{c}\left(\tau_{0}+\frac{d_{m}}{c}\right)\right).$$

Then, after taking the second partial derivatives of L, we obtain

(10) ∂2L∂x∂y=−Re∑m=1M∑n=0N−12σm2x−xmcdmy−ymcdmjωcsm(n)+spm(n)2−sm(n)−um(n)*f6n,m,αxmdm2,

where $f_{6}\left(n,m,\alpha \right)$ is a deterministic function without an impact on the final result (as explained in Appendix A). These expressions depend on $u_{m}\left(n\right)$, which we treat as random variables (not their realizations). After applying the expectation operator to them, we get FIM elements of the form

(11) $$I_{23}=-E\frac{\partial^{2}L}{\partial x\partial y}=\beta \sum_{m=1}^{M}\frac{2}{\sigma_{m}^{2}}\frac{\left(x-x_{m}\right)\left(y-y_{m}\right)}{c^{2}d_{m}^{2}},$$

where the multiplication factor is given by

(12) $$\beta =\sum_{n=0}^{N-1}{\left|j\omega_{c}s\left(n\right)+s^{\prime }\left(n\right)\right|}^{2}$$

and is common to all of the FIM elements. It is interesting to note that the first term in β takes into account the impact of the information in the carrier phase on the error bound, whereas the second term (the derivative of the sequence, $s^{\prime }\left(\cdot \right)$) takes into account the bandwidth. Finally, we need to invert the FIM. The elements on the main diagonal of ${\mathrm{FIM}}^{-1}$ are the error bounds for $\tau_{0}$, *x*, *y*, and *z*, in that order. The bound on the mean squared localization error is then the sum of the elements (2,2), (3,3), and (4,4) of ${\mathrm{FIM}}^{-1}$ for 3D and the sum of (2,2) and (3,3) for 2D localization. Appendix A provides the steps of this derivation in more detail.

Let ${\mathrm{SNR}}_{0}$ be the SNR of a channel whose antenna is 1m away from the transmitter. Based on the derived expressions for the FIM elements, we obtain that the CRB approximately depends on ${\mathrm{SNR}}_{0}$, the carrier frequency, and the number of samples is as follows:

(13) $$\mathrm{CRB}∼1/{\mathrm{SNR}}_{0},$$

(14) $$\mathrm{CRB}∼1/\omega_{c}^{2},$$

(15) CRB∼1/N.

### 3.2. Random Gaussian Sequences (Non-Cooperative Transmitter)

This case corresponds to a scenario where a base station tries to localize a (possibly non-cooperative) terminal transmitting a signal unknown to the base station (probably with a compact spectrum, e.g., OFDM). We reiterate that in our derivation for random sequences, the beginning of the period of the transmitted sequence, $\tau_{0}$, is unknown, but irrelevant for the localization. The reason is that a time-shifted white Gaussian process has the same statistical properties as its version without a time shift. The CRBs derived here are approximate, since the signal is not periodic with period *N* and the DFT-based time shift is cyclic.

Contrary to the scenario with known sequences, here, all of the MN-acquired time samples are dependent in pairs, which makes it inconvenient to form the joint distribution. Namely, if $\tau_{0}+\tau_{m}$ in (1) were an integer, say 17, then $s_{m}\left(0\right)$, as well as $u_{m}\left(0\right)$ in (2), would depend only on s(17). However, in reality, $\tau_{0}+\tau_{m}$ is not an integer, but say 17.439, and therefore $u_{m}\left(0\right)$ depends on all of s(0),s(1),⋯,s(N−1) (s(n) being random variables and $u_{m}\left(0\right)$ being a function of them). The same is true for $u_{m}\left(1\right),u_{m}\left(2\right),\cdots ,u_{m}\left(N-1\right)$ (for that particular channel *m*)—each of them depends on all s(0),s(1),⋯,s(N−1). It would be easier if we had groups of variables such that the groups are independent from each other. Then, the total joint PDF would be a simple product of the PDFs of each group individually.

An idea for addressing this is to transform the received signals into the frequency domain (using DFT). We perform the DFT on the time domain vector $\left[u_{m}\left(0\right),u_{m}\left(1\right),\cdots ,u_{m}\left(N-1\right)\right]$ to get a vector of DFT components, and we repeat this for each Rx channel *m*, $m\in \left\{1,2,\cdots ,M\right\}$. This way, we obtain a new statistical sample, which contains MN frequency samples, instead of the old one, which contains MN time samples, but the amount of information in the new one and in the old one is the same (they are equivalent). Next, at a given frequency with index *k*, we can group the *k*-th DFT component from each of the Rx channels into an *M*-element vector $\left[{\tilde{u}}_{1}\left(k\right),{\tilde{u}}_{2}\left(k\right),\cdots ,{\tilde{u}}_{M}\left(k\right)\right]$. We show that these *N* groups (random vectors) are independent from each other (exactly what we were looking for). Finally, all that remains is to find the PDF of each group and calculate their product. We proceed by explaining these steps in more detail in the following text. As a convention, we will use the symbol x˜ to denote DFT spectra.

The DFT spectra of the signal and noises are

(16) $$\begin{array}{cc} \tilde{s}\left(k\right) & =F\left(s(n)\right)=\sum_{n=0}^{N-1}s\left(n\right)\exp\left(-j2\pi \frac{kn}{N}\right), \end{array}$$

(17) $$\begin{array}{cc} {\tilde{u}}_{m}\left(k\right) & =F\left(u_{m}\left(n\right)\right),\;{\tilde{w}}_{m}\left(k\right)=F\left(w_{m}\left(n\right)\right), \end{array}$$

where F denotes the DFT operator.

In order to express time shifts in the spectral domain realistically, let the discrete frequency be in the range $k\in \left\{-\frac{N}{2},-\frac{N}{2}+1,\cdots ,\frac{N}{2}-1\right\}$.

The reason for this choice is that the DFT components in the complex baseband domain need to correspond correctly to the RF frequencies in the range $\left[\nu_{c}-B/2,\nu_{c}+B/2\right]$. If we chose *k* to be in $\left\{0,1,\cdots ,N-1\right\}$ (as in classical signal processing), that would correspond to the frequencies in the range $\left[\nu_{c},\nu_{c}+B\right]$, which would be in conflict with the model. Since $s_{m}\left(n\right)=\exp\left(-j\omega_{c}d_{m}/c\right)s\left(n-d_{m}/c\right)$, relying on the properties of the DFT operator defined in (16), we have

(18) $$\begin{array}{cc} {\tilde{s}}_{m}\left(k\right) & =\tilde{s}\left(k\right)\exp\left(-j\left(\omega_{c}+2\pi k/N\right)d_{m}/c\right), \end{array}$$

(19) $$\begin{array}{cc} {\tilde{u}}_{m}\left(k\right) & ={\tilde{s}}_{m}\left(k\right)+{\tilde{w}}_{m}\left(k\right), \end{array}$$

where ${\tilde{s}}_{m}\left(k\right)=F\left(s_{m}\left(n\right)\right)$. Since the noise signals $w_{m}\left(n\right)$ and the unknown useful signal s(n) are white (uncorrelated time samples) stationary complex Gaussian random processes, their DFT components, ${\tilde{w}}_{m}\left(k\right)$ and $\tilde{s}\left(k\right)$, are also i.i.d. (independent and identically distributed) complex Gaussian [39]. The fact that the samples at different frequencies are independent is a very convenient property when we need to find the joint PDF—a property we do not have in the time domain. Note that the time samples s(n) and $w_{m}\left(n\right)$ are independent because, in the model, we consider that the sampling is performed at the Nyquist frequency. This, however, does not restrict the analysis in this paper to such systems. Even if a system performs oversampling (which has some advantages), it can reduce the sampling frequency to the Nyquist frequency later in the digital domain. Regardless, the amount of information is the same (in the band of interest), which is important for the analysis of precision. Therefore, the model is general (it holds for both types of systems). To summarize in a more formal way, the properties of s(n) and $w_{m}\left(n\right)$ imply that Res˜(k), Ims˜(k), Res˜m(k), and Ims˜m(k) have Gaussian distributions, i.e., Res˜m(k),Ims˜m(k)∼N0,Nσs2/2, and Rew˜m(k),Imw˜m(k)∼N0,Nσm2/2. Furthermore, the processes ${\tilde{s}}_{m}$ and ${\tilde{w}}_{m}$ are also independent, as are the random variables ${\tilde{s}}_{m}\left(k_{1}\right)$ and ${\tilde{s}}_{m}\left(k_{2}\right)$ (and similarly ${\tilde{w}}_{m}\left(k_{1}\right)$ and ${\tilde{w}}_{m}\left(k_{2}\right)$) for $k_{1}\neq k_{2}$. Finally, the real components are independent from the imaginary components.

Instead of having time samples in the statistical sample, we can compute and use the DFT samples of the received signals. So, the entire statistical sample (containing MN complex scalars from the frequency domain) is

(20) $$\tilde{u}=\;\left[\begin{array}{cccc} {\tilde{u}}_{1}\left(-N/2\right) & {\tilde{u}}_{1}\left(-N/2+1\right) & \cdots & {\tilde{u}}_{1}\left(N/2-1\right)\\ \vdots & \vdots & \ddots & \vdots \\ {\tilde{u}}_{M}\left(-N/2\right) & {\tilde{u}}_{M}\left(-N/2+1\right) & \cdots & {\tilde{u}}_{M}\left(N/2-1\right) \end{array}\right].$$

Note that the *m*-th row of the matrix $\tilde{u}$ is the DFT spectrum of the signal from the *m*-th channel and that the columns (viewed as random vectors) are independent from each other. For the PDF of $\tilde{u}$, we can write

(21) $$p\left(\tilde{u}\right)=\prod_{k=-N/2}^{N/2-1}p\left({\tilde{u}}_{1}\left(k\right),{\tilde{u}}_{2}\left(k\right),\cdots ,{\tilde{u}}_{M}\left(k\right)\right),$$

because the random variables for different frequencies are independent [39]. Also, we have

(22) $$\begin{array}{cc} p\left(\tilde{s}\left(k\right)\right) & =\frac{1}{\pi N\sigma_{s}^{2}}\exp\left(-\frac{1}{N\sigma_{s}^{2}}{\left|\tilde{s}\left(k\right)\right|}^{2}\right), \end{array}$$

(23) $$\begin{array}{cc} p\left({\tilde{w}}_{m}\left(k\right)\right) & =\frac{1}{\pi N\sigma_{m}^{2}}\exp\left(-\frac{1}{N\sigma_{m}^{2}}{\left|{\tilde{w}}_{m}\left(k\right)\right|}^{2}\right), \end{array}$$

where $\tilde{s}\left(k\right),{\tilde{w}}_{1}\left(k\right),\cdots ,{\tilde{w}}_{M}\left(k\right)$ are independent.

In Appendix B, first we show that we can write the loglikelihood as

(24) L=lnpu˜=NlnγπA+∑k=−N/2N/2−11AN2∑m=1MCm+jDmσm22−∑m=1MCm+jDm2Nσm2∑m=1MCm+jDmσm22,

where $\gamma =\frac{1}{\pi N\sigma_{s}^{2}}\prod_{m=1}^{M}\frac{1}{\pi N\sigma_{m}^{2}}$, $A=\frac{1}{N\sigma_{s}^{2}}+\sum_{m=1}^{M}\frac{1}{N\sigma_{m}^{2}}$, and where $C_{m}$ and $D_{m}$ are real and

(25) $$C_{m}+jD_{m}={\tilde{u}}_{m}\left(k\right)\exp\left(j\left(\omega_{c}+2\pi k/N\right)d_{m}/c\right).$$

We note that γ and *A* do not depend on α.

The FIM of the parameters is given by

(26) $$I\left(\alpha \right)={\left[I_{ij}\right]}_{3\times 3},\;\;I_{ij}=-E\left(\frac{\partial^{2}L}{\partial \alpha_{i}\partial \alpha_{j}}\right)=I_{ji}.$$

Taking the first partial derivatives of L with respect to the unknown parameters given in α, we get expressions of the form

(27) ∂L∂x=2AN2Re∑k=−N/2N/2−1∑m=1M1σm2u˜m*(k)×exp−jωc+2πkNdmc∑m=1M×∑m=1M1σm2u˜m(k)expjωc+2πkNdmc×jωc+2πkNx−xmcdm.

Then, taking the second partial derivatives yields expressions of the form

(28) ∂2L∂x2=2AN2×Re∑k=−N/2N/2−1∑m=1M1σm2u˜m(k)×ωc+2πkN×expjωc+2πkNdmcx−xmcdm2+∑m=1M1σm2×u˜m*(k)×exp−jωc+2πkNdmc×∑m=1M1σm2×u˜m(k)×jωc+2πkN×expjωc+2πkNdmc×jωc+2πkN×x−xmcdm2+1cdm−(x−xm)2cdm3xmdm2∑m=1M2.

Next, we apply the expectation operator to the second derivatives (treating the DFT components ${\tilde{u}}_{m}\left(k\right)$ as random variables) to get the FIM elements, of the form

(29) $$I_{11}=\beta \sum_{m=1}^{M}\sum_{p=1}^{M}\frac{1}{\sigma_{m}^{2}\sigma_{p}^{2}}\frac{x-x_{p}}{cd_{p}}\left(\frac{x-x_{p}}{cd_{p}}-\frac{x-x_{m}}{cd_{m}}\right),$$

where the multiplication factor

(30) $$\beta =\frac{2\sigma_{s}^{2}}{\frac{1}{\sigma_{s}^{2}}+\sum_{m=1}^{M}\frac{1}{\sigma_{m}^{2}}}\left(N\omega_{c}^{2}-2\pi \omega_{c}+\frac{N^{2}+2}{3N}\pi^{2}\right),$$

is common to all of the FIM elements. Since the FIM in the random sequence case does not have a row/column that corresponds to $\tau_{0}$, the bound on the mean squared localization error is simply the trace of ${\mathrm{FIM}}^{-1}$ for 3D and the sum of the elements (1,1) and (2,2) for 2D localization. Appendix B provides the steps of this derivation in more detail.

Based on the expressions for the FIM elements, the CRB approximately depends on ${\mathrm{SNR}}_{0}$, the carrier frequency, and the number of samples as follows:

(31) $$\mathrm{CRB}∼1/{\mathrm{SNR}}_{0}^{2},\;\mathrm{low}\;\mathrm{SNRs},$$

(32) $$\mathrm{CRB}∼1/{\mathrm{SNR}}_{0},\;\mathrm{high}\;\mathrm{SNRs},$$

(33) $$\mathrm{CRB}∼1/\omega_{c}^{2},$$

(34) CRB∼1/N.

## 4. Discussion

**Figure 2:**
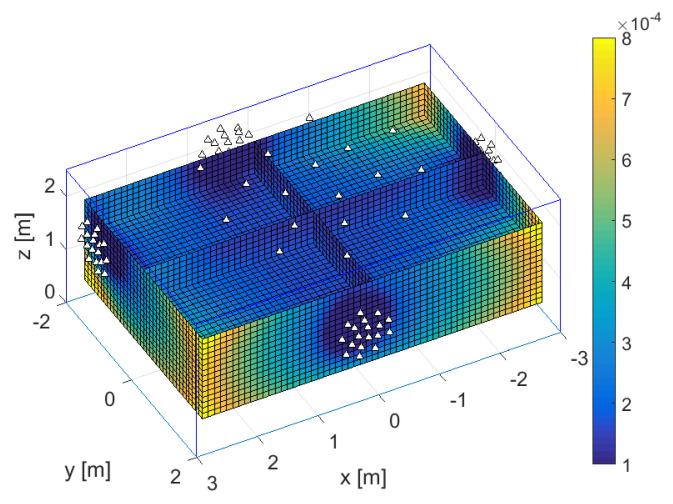
$\sqrt{\mathrm{CRB}}/\lambda_{c}$ of 3D localization inside a room of size 6m×4m×2.5m with 18 antennas on each wall and the ceiling, all marked with triangles. The obtained CRB is for a random Gaussian sequence and parameters ${\mathrm{SNR}}_{0}=25\;\mathrm{dB}$, N=1024, $f_{c}=60\;\mathrm{GHz}/100\;\mathrm{MHz}$.

In this section, we present experimental results that provide further insights on the localization performance of the studied system. Figure 2 shows a 3D slice representation of the CRB for 3D localization inside a room of size 6m×4m×2.5m, with 18 antennas on each wall and the ceiling (90 in total), marked with triangles, for ${\mathrm{SNR}}_{0}=25\;\mathrm{dB}$, N=1024, $f_{c}=60\;\mathrm{GHz}/100\;\mathrm{MHz}$, and a random Gaussian sequence. For convenience, the parameters are also displayed in Table 2, case 1. In the figure, we plotted $\sqrt{\mathrm{CRB}}/\lambda_{c}$ as a function of the 3D space, where $\lambda_{c}=c/f_{c}=\tilde{c}/\nu_{c}$ is the carrier wavelength. We imagined that a distributed array would come in prefabricated panels with subarrays of antennas for easy installation. So, we modeled four smaller subarrays (but still with inter-antenna distances considerably larger than $\lambda_{c}/2$) on the walls, and a larger one (with a larger aperture) on the ceiling.

Note that the cuboid frame is only a rough model of the room—There would be furniture and people inside and the walls are not expected to be completely flat in practice. Furthermore, they would not be ideal reflective surfaces, but the wave would penetrate the walls to an unknown depth, depending on their conductance and other parameters, changing the carrier phase by a significant amount. Using the information in NLoS components in coherent localization would be much more challenging than LoS components, because ray-tracing-based localization algorithms would have to know the depth, whereas fingerprinting-based methods might require the frequent retraining of their databases (since variations in the phases have to be taken into account here). However, this remains an interesting topic for plausibility studies in future research—Could the obstacles be anchored in space with high enough precision as well (as the Rx antennas are) to enable the gains of coherent localization from their reflected components? This is outside the scope of this paper, so we discard the NLoS components in this error-bound analysis.

**Figure 3:**
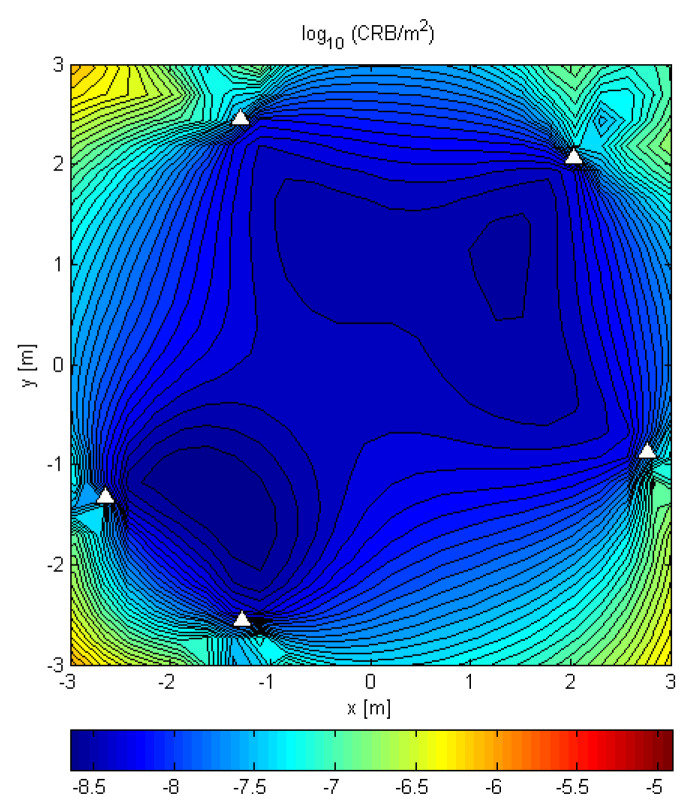
CRB for 2D localization by five randomly placed antennas in the plain of the array. The locations of the antennas are marked by triangles. The obtained CRB is for a random Gaussian sequence and parameters ${\mathrm{SNR}}_{0}=10\;\mathrm{dB}$ and N=64.

Figure 3 shows the CRB for a distributed array with five randomly placed antennas for 2D localization in the plain of the array, for ${\mathrm{SNR}}_{0}=10\;\mathrm{dB}$, N=64, and a random Gaussian sequence (Table 2, case 2). Since the CRB is much lower inside the array aperture, this suggests that a distributed array should be placed in such a way that its aperture encompasses the area where one expects the transmitters.

It is worth noting that both of these example antenna array geometries were chosen to be irregular. An impact on localization performance could be seen as analogous to classical arrays, where nonuniform or sparse arrays perform better than uniform ones for a given number of antennas. In [40], a uniform antenna array with a large number of elements was optimized by reducing the number of used elements greatly (a more cost-effective solution), effectively creating a sparse array with performance comparable to that of the original array. The optimization of an array geometry is also out of scope here, but is an interesting topic for further research. Our idea is that this analysis would also be applicable to geometries that would be set up as ad hoc and then the antenna positions measured.

**Figure 4:**
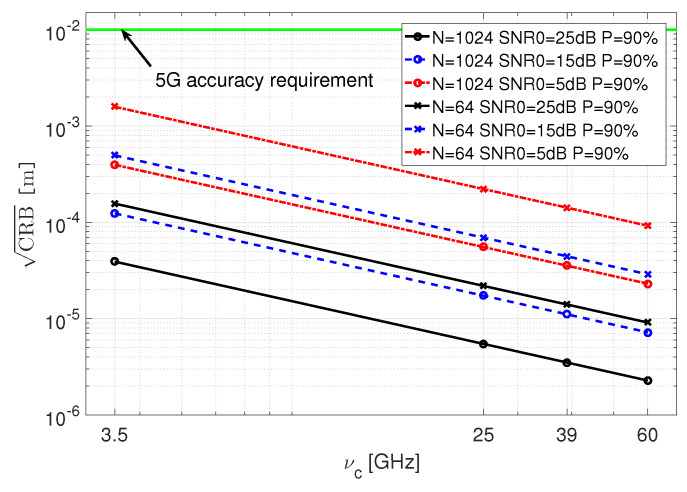
The 0.9 quantile of $\sqrt{\mathrm{CRB}}$ vs. $\nu_{c}$ for a Gaussian sequence and B=100MHz, for different ${\mathrm{SNR}}_{0}$ and *N*.

In Figure 4, we present how the 90% quantile of $\sqrt{\mathrm{CRB}}$ depends on $\nu_{c}$, ${\mathrm{SNR}}_{0}$, and *N*, for a random Gaussian sequence (Table 2, case 3). The plots show how the accuracy of localization improves with frequency, *N*, and SNR${}_{0}$. They also demonstrate the advantage of using mmWave because the increased frequency of the carrier allows for higher localization accuracy. The results for known sequences are similar.

**Figure 5:**
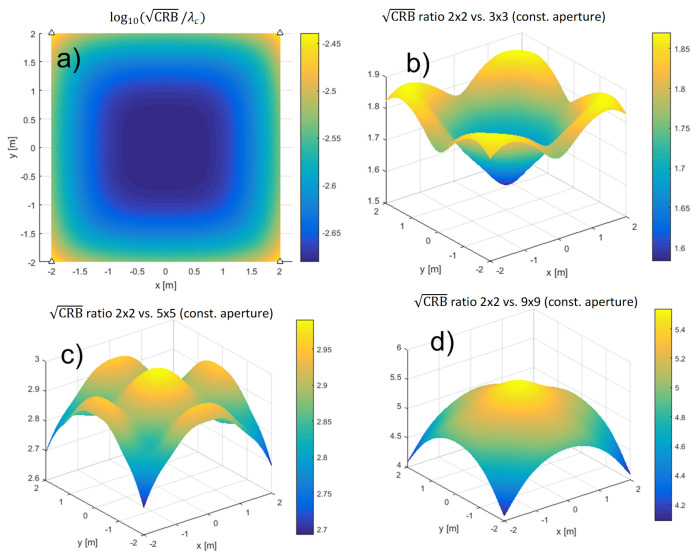
The CRBs for different array sizes in the constant aperture scenario. The square root of the CRB, normalized by the carrier wavelength, for the 2×2 array is shown in (**a**). The ratios of 2×2 on one hand and 3×3, 5×5, and 9×9 on the other are shown in (**b**), (**c**), and (**d**), respectively.

**Figure 6:**
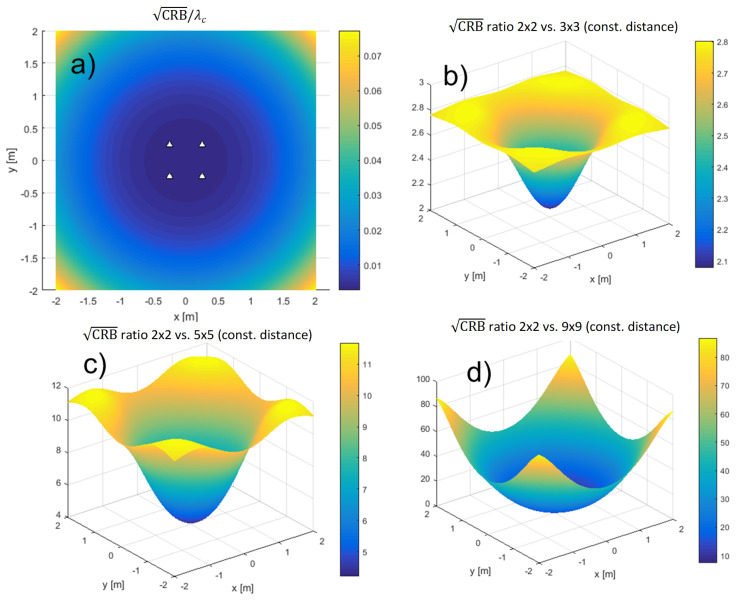
The CRBs for different array sizes in the constant antenna distance scenario. The square root of the CRB, normalized by the carrier wavelength, for the 2×2 array is shown in (**a**). The ratios of 2×2 on one hand and 3×3, 5×5, and 9×9 on the other are shown in (**b**), (**c**), and (**d**), respectively.

It is also worth investigating how the CRBs depend on the number of antennas, *M*. Since the effect of relative placement of antennas to each other and the effect of increasing *M* are coupled, we have chosen to evaluate the CRBs for a uniform square distributed array with 2×2, 3×3, 5×5, and 9×9 antennas (so $M\in \left\{4,9,25,81\right\}$). The arrays were attached to the ceiling of a room and the CRBs were evaluated in a square of size 4m×4m and centered 1.2m directly below the array, in a horizontal plane, for ${\mathrm{SNR}}_{0}=15\;\mathrm{dB}$, N=1024, $f_{c}=60\;\mathrm{GHz}/100\;\mathrm{MHz}$ (Table 2, case 4). We analyzed a constant aperture and a constant antenna distance scenario. In the former, the array’s aperture was kept at 4m×4m, whereas in the latter, the adjacent antennas were retained at 0.5m from each other, effectively making the 9×9 arrays identical in both setups. Figure 5 compares the CRBs of the arrays in the constant aperture and Figure 6, in the constant antenna distance scenario.

**Figure 7:**
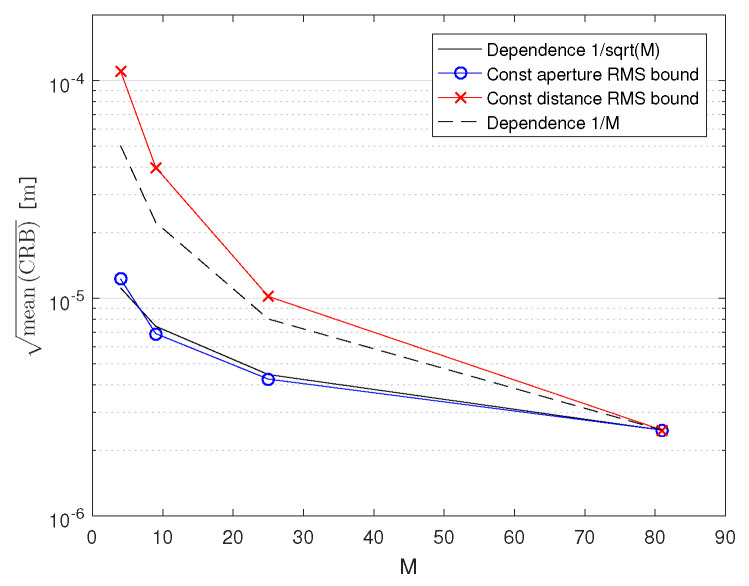
Dependence of the CRB on the number of antennas, *M*.

When we average the CRBs over the possible locations of the transmitter in the 4m×4m square of the experiment, we obtain the results depicted in Figure 7. They show how the CRBs change with *M* and comparing them with the ideal ∼1/*M* and ∼1/$M^{2}$ dependencies. This shows how much increasing the number of antennas improves the accuracy of localization.

**Figure 8:**
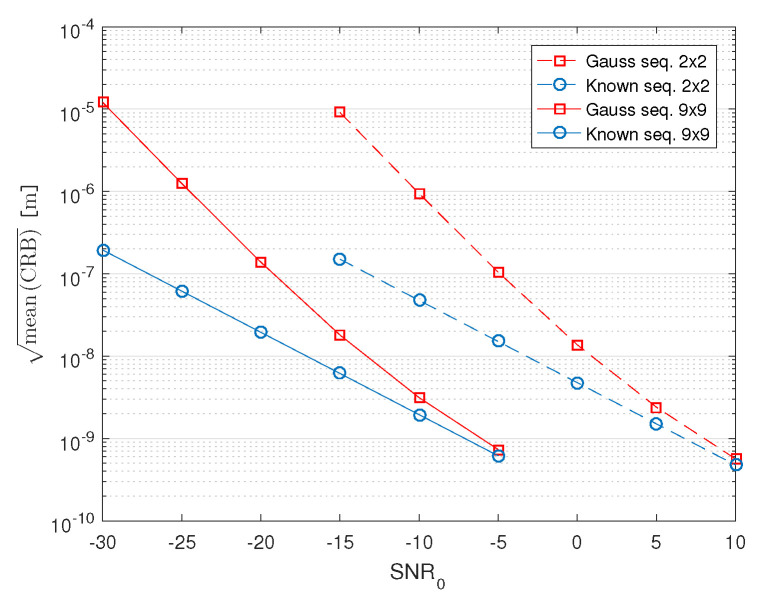
Dependence of the CRB, averaged over space, on ${\mathrm{SNR}}_{0}$.

We have also evaluated the CRB curves as a function of ${\mathrm{SNR}}_{0}$ (recall that ${\mathrm{SNR}}_{0}$ is the SNR of a channel whose antenna is 1 m away from the transmitter) for random Gaussian sequences and known sequences (Table 2, case 5). Again, the results are obtained by averaging the CRBs for locations of the transmitter over locations in the 4m×4m square of the experiment. The results for the 2×2 and 9×9 arrays are shown in Figure 8. They suggest that increasing the number of antennas from 4 to 81 produces a gain of approximately 15dB. The results also show the advantage of using mmWave massive MIMO systems because the localization can successfully be performed in low SNR conditions (low transmitted power in the mmWave range), even for unknown sequences (non-cooperative scenarios). In [18], the authors present additional results for CRBs and localization algorithms evaluated for different sets of parameters, including different array geometries.

Finally, we point out that in coherent direct localization, besides the main lobe of the criterion function of the localization algorithm, there usually exists a number of high sidelobes (the space between them is on the order of the carrier wavelength), so that some estimates are placed on these lobes, degrading the absolute error. This is called the (integer wavelength) ambiguity problem (for further details, please see [18]). However, for distributed beamforming (which is one of the important applications of coherent localization), this absolute error is irrelevant, whereas the error within the main lobe (the distance of its local maximum to the true location) determines its performance. This error is much smaller than the absolute one and is reflected in the CRB (which describes only local behavior). For this reason, the ambiguity problem is outside the scope of this paper. Some possible solutions, though, are mentioned in [18].

## 5. Conclusions

In this paper, we have addressed the performance limits of direct wideband coherent 3D localization in distributed mmWave massive MIMO for 5G cellular systems. In the derivation of the limits, we assume only the presence of spatially coherent line-of-sight signal components. We also assume collocated time- and phase-synchronized receiving front-ends with antennas distributed at known locations. The results for known and random Gaussian signals show that the CRB is inversely proportional to the squared carrier frequency. For a typical indoor setting and realistic system parameters (M=5×18, ${\mathrm{SNR}}_{0}=25\;\mathrm{dB}$, N=1024), the square root of the CRB is smaller than $\lambda_{c}/1000$ in 90% of the space. These results suggest important advantages of massive MIMO for 5G systems. Namely, one can preprocess the signals at the base station array to focus energy at the selected user antenna in the downlink and, conversely, to increase the gain in the uplink. This shifts the focus from location-based services to the location aided communication concept. This entails that coherent localization in LoS scenarios, typical for small cells in mmWave range, has the potential to improve the capacity considerably and the overall performance of 5G massive MIMO systems with distributed antennas. The results also show that the studied systems are especially suited for coherent localization due to high accuracy and the ability to perform localization in low SNR conditions (low radiated power), even with non-cooperative transmitters. The ambiguity problem of coherent localization can be solved in a number of ways, e.g, by optimizing the antenna geometry or by using signals from antenna subarrays distributed in the cell.

Finally, we provide some topics for future research. In practice, the propagation conditions inside a cell may vary over time, so it is desirable for a localization system to implement different methods (with different strengths and weaknesses), such as RSS and coherent ones. It would be valuable to compare their performance and design schemes that activate appropriate methods based on the detected conditions.

Furthermore, optimization of antenna array geometries based on different criteria is of interest. One criterion is the accuracy, but the main accuracy gain seems to be achieved by placing the array so that the transmitters are inside its aperture. Another criterion is the reduction of the ambiguity problem, which can easily happen in coherent localization when the number of antennas is small. This is of interest in applications where absolute error is important (traffic collision prevention, automated robot and drone movement control).

A challenging direction of research is an error analysis in conditions with more pronounced NLoS components and the possibility to leverage information in strong reflections to improve coherent localization.

The exploration of a fair comparison between the error bounds of RSS (received signal strength; i.e., amplitude only) and coherent localization (from this paper) is also important. RSS localization puts no additional requirements on the hardware. To make the comparison fair, it would have to be carried out in conditions suitable for both types. Namely, RSS localization is robust against synchronization errors and a lack of spatial coherence in the medium. On the other hand, coherent localization is robust against deviations in the received power from a law with a given path loss exponent. This is especially important for 3D localization, where not all of the antenna polarizations would be aligned, so that it could even happen that an Rx antenna closer to the Tx has a weaker received signal power than another Rx antenna farther away.

Another interesting topic for further research is the possibility of using reconfigurable intelligent surfaces (RIS) to aid coherent localization for increased accuracy (the use of a RIS in non-coherent localization was already reported in [19]). Namely, if a localization system could measure very accurately the position of a RIS and if it controls (or knows) the phase changes that the RIS induces in the signal component, it reflects from a transmitter (that needs to be localized). Then, a coherent localization algorithm might be able to use such an NLoS component in addition to the LoS component for better performance (especially if the transmitter is well outside the aperture of the Rx antenna array). The paper [41] presents challenges and opportunities for using RISs in localization.

## Figures and Tables

**Table 1:** Symbols and their meaning.

Symbol	—	Meaning
Tx	—	Transmitter/transmitting/transmitted
Rx	—	Receiver/receiving/received
*M*	—	Number of Rx antennas and (front-end) channels
*N*	—	Number of acquired complex baseband samples in each Rx channel
$m\in \left\{1,2\cdots M\right\}$	—	Index of an Rx channel/antenna
$n\in \left\{0,1\cdots N-1\right\}$	—	Time index in the digital domain (discrete time variable)
t∈ℝ	—	Normalized continuous time variable (the unit is one sampling period, i.e., [sample])
*k*	—	DFT (discrete Fourier transform) frequency index
r=(x,y,z)	—	(Unknown) location of the Tx antenna
$r_{m}=\left(x_{m},y_{m},z_{m}\right)$	—	(Known) location of the *m*-th Rx antenna
$d_{m}=∥r-r_{m}∥$	—	Distance between $r_{m}$ and r
*B*	—	Signal bandwidth in [Hz]
$\nu_{s}$	—	Sampling frequency in [Hz]
$\nu_{c}$	—	Carrier frequency in [Hz]
$f_{c}$	—	Normalized carrier frequency in [cycle/sample]
$\omega_{c}$	—	Normalized angular carrier frequency in [rad/sample]
$\lambda_{c}$	—	Carrier wavelength in [m]
$\tilde{c}=3\times 10^{8}[m/s$]	—	Speed of propagation in [m/s]
*c*	—	Normalized speed of propagation in [m/sample]
$\tau_{0}\in R$	—	Time shift between the Tx *t*-axis and the Rx system’s *t*-axis (the lack of time-synchronization)
$\tau_{m}=d_{m}/c\in R$	—	Propagation time from Tx to ${\mathrm{Rx}}_{m}$
s(n)	—	Complex baseband transmitter sequence
s(t)	—	Continuous waveform corresponding to the transmitter sequence
$s^{\prime }\left(n\right)$	—	The first derivative of the waveform s(t) evaluated at t=n
$u_{m}\left(n\right)$	—	Received (noisy) signal in channel *m*
$w_{m}\left(n\right)$	—	Noise in channel *m*
$\sigma_{m}^{2}$	—	Variance of $w_{m}\left(n\right)$
$a_{m}$	—	Amplitude (attenuation) factor for the line-of-sight (LoS) component in channel *m*
$s_{m}\left(n\right)$	—	The LoS component at the Rx antenna *m* (without the amplitude factor)
${\mathrm{SNR}}_{m}$	—	Signal-to-noise (SNR) ratio in channel *m*
${\mathrm{SNR}}_{0}$	—	Signal-to-noise ratio in an imagined channel if its antenna were 1m away from the Tx (the referent SNR)
α	—	Vector of unknown parameters
$I\left(\alpha \right)$	—	Fisher information matrix (FIM)
$I_{ij}$	—	Element (i,j) of the FIM
$p\left(u_{m}\left(n\right);\alpha \right)$	—	Probability density function (PDF) of $u_{m}\left(n\right)$, given the value of α
L	—	Log-likelihood function
$\tilde{s}\left(k\right)$, ${\tilde{u}}_{m}\left(k\right)$, ${\tilde{w}}_{m}\left(k\right)$, …	—	DFT spectrum of s(n), $u_{m}\left(n\right)$, $w_{m}\left(n\right)$, …
I, u, *…*	—	Matrices and column-vectors/row-vectors
N	—	Gaussian (normal) distribution
CN	—	Circularly symmetric complex Gaussian distribution
x*	—	Conjugation
Re	—	Real part
Im	—	Imaginary part
E	—	Expectation operator
Var	—	Variance

**Table 2:** Parameters used for the numerical results.

Case	${\mathrm{SNR}}_{0}$	*N*	Sequence	$\nu_{c}$	$\nu_{s}$
1 (90-ant. array, 3D loc.)	25dB	1024	random	60GHz	100MHz
2 (5-ant. array, 2D loc.)	10dB	64	random	60GHz	100MHz
3 (5-ant. array, 2D loc.)	variable	variable	random	variable	100MHz
4 (square arrays)	15dB	1024	known	60GHz	100MHz
5 (square arrays)	variable	1024	both	60GHz	100MHz

## Data Availability

Not applicable.

## Abbreviations

The following abbreviations are used in the manuscript:

2D	2-dimensional
3D	3-dimensional
5G	Fifth generation
6G	Sixth generation
BPU	Baseband processing unit
C-RAN	Cloud radio access networks
CDMA	Code-division multiple access
CoMP	Coordinated multipoint
CRB	Cramér-Rao bound
DFT	Discrete Fourier transform
FIM	Fisher information matrix
i.i.d.	Independent and identically distributed
IQ	In-phase-quadrature-phase
LAC	Location aided communication
LoS	Line-of-sight
MIMO	Multiple-input multiple-output
mmWave	Millimeter-wave
NLoS	Non-line-of-sight
PDF	Probability density function
RAU	Remote antenna unit
RF	Radio frequency
RFID	Radio frequency identification
RFoF	Radio-frequency-over-fiber
RIS	Reconfigurable intelligent surface
RSS	Received signal strength
SNR	Signal-to-noise ratio
TDoA	Time difference of arrival
ToA	Time of arrival

## Appendix A. CRB Derivation-Known Sequences

We rewrite the loglikelihood function from (6), $L=\ln p\left(u|\alpha \right)$, i.e.,

(A1) $$L=-N\sum_{m=1}^{M}\ln\left(\pi \sigma_{m}^{2}\right)-\sum_{m=1}^{M}\sum_{n=0}^{N-1}\frac{1}{\sigma_{m}^{2}}{\left|s_{m}\left(n\right)-u_{m}\left(n\right)\right|}^{2}.$$

The FIM is given by

(A2) $$I\left(\alpha \right)={\left[I_{ij}\right]}_{4\times 4}\;I_{ij}=-E\frac{\partial^{2}L}{\partial \alpha_{i}\partial \alpha_{j}}=I_{ji}.$$

In the derivation, we use the identity ∂∂αz2=2Re(z*∂z∂α), where (·)* denotes complex conjugation. Note also that since

(A3) $$d_{m}=\sqrt{{\left(x-x_{m}\right)}^{2}+{\left(y-y_{m}\right)}^{2}+{\left(z-z_{m}\right)}^{2}}$$

we can write

(A4) $$\frac{\partial d_{m}}{\partial x}=\frac{x-x_{m}}{d_{m}},\;\frac{\partial d_{m}}{\partial y}=\frac{y-y_{m}}{d_{m}},\;\frac{\partial d_{m}}{\partial z}=\frac{z-z_{m}}{d_{m}}$$

Define

(A5) $$s_{pm}\left(n\right)=s^{\prime }\left(n-\tau_{0}-\frac{d_{m}}{c}\right)\times \exp\left(-j\omega_{c}\left(\tau_{0}+\frac{d_{m}}{c}\right)\right)$$

where (·)′ denotes the first derivative. Then, the first derivatives of the loglikelihood are

(A6) ∂L∂τ0=Re∑m=1M∑n=0N−12σm2sm(n)−um(n)*×jωcsm(n)+spm(n),

(A7) ∂L∂x=Re∑m=1M∑n=0N−12σm2sm(n)−um(n)*×x−xmcdmjωcsm(n)+spm(n),

(A8) ∂L∂y=Re∑m=1M∑n=0N−12σm2sm(n)−um(n)*×y−ymcdmjωcsm(n)+spm(n),

(A9) ∂L∂z=Re∑m=1M∑n=0N−12σm2sm(n)−um(n)*×z−zmcdmjωcsm(n)+spm(n).

If we define $P_{m}={\left|j\omega_{c}s_{m}\left(n\right)+s_{pm}\left(n\right)\right|}^{2}$, then for the second derivatives we obtain

(A10) ∂2L∂τ02=−Re∑m=1M∑n=0N−12σm21*Pm−sm(n)−um(n)*f1n,m,α,

(A11) ∂2L∂τ0∂x=−Re∑m=1M∑n=0N−12σm2x−xmcdmPm−sm(n)−um(n)*f2n,m,αxmdm,

(A12) ∂2L∂τ0∂y=−Re∑m=1M∑n=0N−12σm2y−ymcdmPm−sm(n)−um(n)*f3n,m,αxmdm,

(A13) ∂2L∂τ0∂z=−Re∑m=1M∑n=0N−12σm2z−zmcdmPm−sm(n)−um(n)*f4n,m,αxmdm,

(A14) ∂2L∂x2=−Re∑m=1M∑n=0N−12σm2x−xmcdm2Pm−sm(n)−um(n)*f5n,m,αxmdm2,

(A15) ∂2L∂x∂y=−Re∑m=1M∑n=0N−12σm2x−xmcdmy−ymcdmPm−sm(n)−um(n)*f6n,m,αxmdm2,

(A16) ∂2L∂x∂z=−Re∑m=1M∑n=0N−12σm2x−xmcdmz−zmcdmPm−sm(n)−um(n)*f7n,m,αxmdm2,

(A17) ∂2L∂y2=−Re∑m=1M∑n=0N−12σm2y−ymcdm2Pm−sm(n)−um(n)*f8n,m,αxmdm2,

(A18) ∂2L∂y∂z=−Re∑m=1M∑n=0N−12σm2y−ymcdmz−zmcdmPm−sm(n)−um(n)*f9n,m,αxmdm2,

(A19) ∂2L∂z2=−Re∑m=1M∑n=0N−12σm2z−zmcdm2Pm−sm(n)−um(n)*f10n,m,αxmdm2,

where $f_{i}\left(n,m,\alpha \right)$, 1≤i≤10, are deterministic functions, whose expressions are not given for brevity. They are all deterministic, and each of them is multiplied by the term ${\left(s_{m}\left(n\right)-u_{m}\left(n\right)\right)}^{*}$ whose expectation is equal to zero, and therefore, their values have no impact on the result in the next step.

Then, for the first element of the FIM we write

$$I_{11}=\sum_{m=1}^{M}\frac{2}{\sigma_{m}^{2}}\sum_{n=0}^{N-1}{\left|j\omega_{c}s_{m}\left(n\right)+s_{pm}\left(n\right)\right|}^{2}.$$

If we define β as

$$\beta =\sum_{n=0}^{N-1}{\left|j\omega_{c}s_{m}\left(n\right)+s_{pm}\left(n\right)\right|}^{2},$$

after some simple derivation, we get

(A20) $$\beta =\sum_{n=0}^{N-1}{\left|j\omega_{c}s\left(n\right)+s^{\prime }\left(n\right)\right|}^{2},$$

because shifting a signal in time does not change its energy. Note that β does not depend on *m*. Then $I_{11}$ and the rest of the FIM elements can be expressed in compact forms respectively as

(A21) $$\begin{array}{cc} I_{11} & =-E\frac{\partial^{2}L}{\partial \tau_{0}^{2}}=\beta \sum_{m=1}^{M}\frac{2}{\sigma_{m}^{2}}, \end{array}$$

(A22) $$\begin{array}{cc} I_{12} & =-E\frac{\partial^{2}L}{\partial \tau_{0}\partial x}=\beta \sum_{m=1}^{M}\frac{2}{\sigma_{m}^{2}}\frac{x-x_{m}}{cd_{m}}, \end{array}$$

(A23) $$\begin{array}{cc} I_{13} & =-E\frac{\partial^{2}L}{\partial \tau_{0}\partial y}=\beta \sum_{m=1}^{M}\frac{2}{\sigma_{m}^{2}}\frac{y-y_{m}}{cd_{m}}, \end{array}$$

(A24) $$\begin{array}{cc} I_{14} & =-E\frac{\partial^{2}L}{\partial \tau_{0}\partial z}=\beta \sum_{m=1}^{M}\frac{2}{\sigma_{m}^{2}}\frac{z-z_{m}}{cd_{m}}, \end{array}$$

(A25) $$\begin{array}{cc} I_{22} & =-E\frac{\partial^{2}L}{\partial x^{2}}=\beta \sum_{m=1}^{M}\frac{2}{\sigma_{m}^{2}}{\left(\frac{x-x_{m}}{cd_{m}}\right)}^{2}, \end{array}$$

(A26) $$\begin{array}{cc} I_{23} & =-E\frac{\partial^{2}L}{\partial x\partial y}=\beta \sum_{m=1}^{M}\frac{2}{\sigma_{m}^{2}}\frac{\left(x-x_{m}\right)\left(y-y_{m}\right)}{c^{2}d_{m}^{2}}, \end{array}$$

(A27) $$\begin{array}{cc} I_{24} & =-E\frac{\partial^{2}L}{\partial x\partial z}=\beta \sum_{m=1}^{M}\frac{2}{\sigma_{m}^{2}}\frac{\left(x-x_{m}\right)\left(z-z_{m}\right)}{c^{2}d_{m}^{2}}, \end{array}$$

(A28) $$\begin{array}{cc} I_{33} & =-E\frac{\partial^{2}L}{\partial y^{2}}=\beta \sum_{m=1}^{M}\frac{2}{\sigma_{m}^{2}}{\left(\frac{y-y_{m}}{cd_{m}}\right)}^{2}, \end{array}$$

(A29) $$\begin{array}{cc} I_{34} & =-E\frac{\partial^{2}L}{\partial y\partial z}=\beta \sum_{m=1}^{M}\frac{2}{\sigma_{m}^{2}}\frac{\left(y-y_{m}\right)\left(z-z_{m}\right)}{c^{2}d_{m}^{2}}, \end{array}$$

(A30) $$\begin{array}{cc} I_{44} & =-E\frac{\partial^{2}L}{\partial z^{2}}=\beta \sum_{m=1}^{M}\frac{2}{\sigma_{m}^{2}}{\left(\frac{z-z_{m}}{cd_{m}}\right)}^{2}. \end{array}$$

The CRBs for the individual components are given by

(A31) $$\begin{array}{cc} \mathrm{Var}{\hat{\tau }}_{0} & \geq {\mathrm{CRB}}_{\tau_{0}}={\left[I{\left(\alpha \right)}^{-1}\right]}_{11}, \end{array}$$

(A32) $$\begin{array}{cc} \mathrm{Var}\hat{x} & \geq {\mathrm{CRB}}_{x}={\left[I{\left(\alpha \right)}^{-1}\right]}_{22}, \end{array}$$

(A33) $$\begin{array}{cc} \mathrm{Var}\hat{y} & \geq {\mathrm{CRB}}_{y}={\left[I{\left(\alpha \right)}^{-1}\right]}_{33}, \end{array}$$

(A34) $$\begin{array}{cc} \mathrm{Var}\hat{z} & \geq {\mathrm{CRB}}_{z}={\left[I{\left(\alpha \right)}^{-1}\right]}_{44}. \end{array}$$

We define by $\Delta r=\sqrt{{\left(\hat{x}-x\right)}^{2}+{\left(\hat{y}-y\right)}^{2}+{\left(\hat{z}-z\right)}^{2}}$ the error in locating the transmitter. For the bound of its mean squared value, we write

(A35) $$\begin{array}{cc} {\mathrm{CRB}}_{\Delta r} & ={\mathrm{CRB}}_{x}+{\mathrm{CRB}}_{y}+{\mathrm{CRB}}_{z}\\ \; & =\frac{K_{22}+K_{33}+K_{44}}{\det I\left(\alpha \right)}, \end{array}$$

where $K_{ij}$ is the cofactor of the $\left(i,j\right)$th element of $I\left(\alpha \right)$. For simplicity of notation, we will call this bound just the (transmitter location) CRB.

If the transmitter’s *z*-coordinate is known (which corresponds to 2D localization), the corresponding FIM, $I^{\left(2D\right)}$, is obtained by removing the last row and column of the FIM for the 3D case, $I\left(\alpha \right)$. Namely,

(A36) $$I^{\left(2D\right)}=\;\left[\begin{array}{ccc} I_{11} & I_{12} & I_{13}\\ I_{12} & I_{22} & I_{23}\\ I_{13} & I_{23} & I_{33} \end{array}\right],$$

and the CRB for 2D localization is

(A37) $$\begin{array}{cc} {\mathrm{CRB}}_{\Delta r}^{\left(2D\right)} & ={\mathrm{CRB}}_{x}^{\left(2D\right)}+{\mathrm{CRB}}_{y}^{\left(2D\right)} \end{array}$$

(A38) $$\begin{array}{cc} \; & =\frac{I_{11}I_{33}-I_{13}^{2}+I_{11}I_{22}-I_{12}^{2}}{\det I^{\left(2D\right)}}. \end{array}$$

Let the quantity ${\mathrm{SNR}}_{0}$ be defined as the SNR value in a channel whose antenna would be 1m away from the transmitter. If the signal power decreases with the square of the distance from the transmitter, and if the noise has equal power across the channels prior to scaling by $1/a_{m}$, starting from the derived expressions above, ${\mathrm{SNR}}_{m}={\mathrm{SNR}}_{0}d_{0}^{2}/d_{m}^{2}$, $d_{0}=1\;m$, and the assumption that $\omega_{c}$ is large in the sense that $\beta \approx \sum_{n=0}^{N-1}{\left|j\omega_{c}s\left(n\right)\right|}^{2}$, it can be shown that the CRBs approximately depend on ${\mathrm{SNR}}_{0}$, the carrier frequency, and the number of samples as follows: (Actually, $\mathrm{CRB}∼1/\nu_{c}^{2}$. So, the expression $\mathrm{CRB}∼1/\omega_{c}^{2}$ holds if *B* is constant, since $\omega_{c}=2\pi \nu_{c}/B$. In addition, the dependence on *B* also exists in *c*.)

(A39) $$\mathrm{CRB}∼1/{\mathrm{SNR}}_{0},$$

(A40) $$\mathrm{CRB}∼1/\omega_{c}^{2},$$

(A41) CRB∼1/N.

## Appendix B. CRB Derivation—Random Gaussian Sequences

We start with writing the joint PDF $p\left({\tilde{u}}_{1}\left(k\right),{\tilde{u}}_{2}\left(k\right),\cdots ,{\tilde{u}}_{M}\left(k\right)\right)$. This random vector is a function of M+1 independent random variables (one for the useful signal, $\tilde{s}\left(k\right)$, and *M* for the noise, ${\tilde{w}}_{m}\left(k\right)$). We obtain the joint PDF by integrating over all the possible values of the signal random variable, $\tilde{s}\left(k\right)$. If we denote Res˜(k)=sR(k) and Ims˜(k)=sI(k), then

(A42) $$\begin{array}{cc} \; & p\left({\tilde{u}}_{1}\left(k\right),{\tilde{u}}_{2}\left(k\right),\cdots ,{\tilde{u}}_{M}\left(k\right)\right)=\int_{-\infty }^{\infty }\int_{-\infty }^{\infty }p\left(s_{R}\left(k\right)\right)p\left(s_{I}\left(k\right)\right)\times p_{{\tilde{w}}_{1}\left(k\right)}\left({\tilde{u}}_{1}\left(k\right)-{\tilde{s}}_{1}\left(k\right)\right)\\ \; & \times p_{{\tilde{w}}_{2}\left(k\right)}\left({\tilde{u}}_{2}\left(k\right)-{\tilde{s}}_{2}\left(k\right)\right)\times \cdots \times p_{{\tilde{w}}_{M}\left(k\right)}\left({\tilde{u}}_{M}\left(k\right)-{\tilde{s}}_{M}\left(k\right)\right)ds_{R}\left(k\right)ds_{I}\left(k\right), \end{array}$$

where $p_{{\tilde{w}}_{m}\left(k\right)}\left({\tilde{u}}_{m}\left(k\right)-{\tilde{s}}_{m}\left(k\right)\right)$ is the value of the PDF of the complex random variable ${\tilde{w}}_{m}\left(k\right)$ evaluated at ${\tilde{u}}_{m}\left(k\right)-{\tilde{s}}_{m}\left(k\right)$. After some rearranging, we obtain

(A43) $$\begin{array}{cc} p\left({\tilde{u}}_{1}\left(k\right),\cdots ,{\tilde{u}}_{M}\left(k\right)\right) & =\gamma \int_{-\infty }^{+\infty }\int_{-\infty }^{+\infty }\exp\left(-\frac{1}{N\sigma_{s}^{2}}{\left|a+jb\right|}^{2}\right)\\ \; & \times \prod_{m=1}^{M}\exp\left(-\frac{1}{N\sigma_{m}^{2}}{\left|a+jb-C_{m}-jD_{m}\right|}^{2}\right)dadb, \end{array}$$

where $C_{m},D_{m}$ (which are real), and γ are defined by

(A44) $$\begin{array}{cc} C_{m}+jD_{m} & ={\tilde{u}}_{m}\left(k\right)\exp\left(+j\left(\omega_{c}+2\pi k/N\right)d_{m}/c\right), \end{array}$$

(A45) $$\begin{array}{cc} \gamma & =\frac{1}{\pi N\sigma_{s}^{2}}\prod_{m=1}^{M}\frac{1}{\pi N\sigma_{m}^{2}}. \end{array}$$

Using $\int_{-\infty }^{+\infty }1/\left(\sqrt{2\pi w}\right)\exp\left(-x^{2}/\left(2w\right)\right)dx=1$ and (21), after some derivation, we get

(A46) pu˜=γπAN×exp∑k=−N/2N/2−11AN2∑m=1MCm+jDmσm22−∑m=1MCm+jDm2Nσm2∑m=1MCm+jDmσm22,

where $A=\frac{1}{N\sigma_{s}^{2}}+\sum_{m=1}^{M}\frac{1}{N\sigma_{m}^{2}}$. Thus, the loglikelihood function is

(A47) L=lnpu˜=NlnγπA+∑k=−N/2N/2−11AN2∑m=1MCm+jDmσm22−∑m=1MCm+jDm2Nσm2∑m=1MCm+jDmσm22.

The FIM is

(A48) $$I\left(\alpha \right)={\left[I_{ij}\right]}_{3\times 3}\;I_{ij}=-E\frac{\partial^{2}L}{\partial \alpha_{i}\partial \alpha_{j}}=I_{ji}.$$

Note that γ, *A* do not depend on α. For the log likelihood, we write further

(A49) $$\begin{array}{cc} L= & N\ln\frac{\gamma \pi }{A}-\sum_{k=-N/2}^{N/2-1}\sum_{m=1}^{M}\frac{1}{N\sigma_{m}^{2}}{\left|{\tilde{u}}_{m}\left(k\right)\right|}^{2}\\ \; & +\frac{1}{AN^{2}}\sum_{k=-N/2}^{N/2-1}{\left|\sum_{m=1}^{M}\frac{1}{\sigma_{m}^{2}}{\tilde{u}}_{m}\left(k\right)\times \exp\left(j\left(\omega_{c}+\frac{2\pi k}{N}\right)\frac{d_{m}}{c}\right)\right|}^{2}. \end{array}$$

Note that the first two terms of L do not depend on α, but the third term does. In the sequel, we use the following identities: ∂∂αz2=2Re(z*∂z∂α), ∂dm∂x=x−xmdm, ∂dm∂y=y−ymdm, and ∂dm∂z=z−zmdm. For the first derivatives of L, we obtain

(A50) ∂L∂x=2AN2Re∑k=−N/2N/2−1∑m=1M1σm2u˜m*(k)×exp−jωc+2πkNdmc∑m=1M×∑m=1M1σm2u˜m(k)expjωc+2πkNdmc×jωc+2πkNx−xmcdm,

(A51) ∂L∂y=2AN2Re∑k=−N/2N/2−1∑m=1M1σm2u˜m*(k)×exp−jωc+2πkNdmc×∑m=1M1σm2Um(k)expjωc+2πkNdmc×jωc+2πkNy−ymcdm,

(A52) ∂L∂z=2AN2Re∑k=−N/2N/2−1∑m=1M1σm2u˜m*(k)×exp−jωc+2πkNdmc×∑m=1M1σm2u˜m(k)expjωc+2πkNdmc×jωc+2πkNz−zmcdm.

The second derivatives of the loglikelihood are

(A53) ∂2L∂x2=2AN2×Re∑k=−N/2N/2−1∑m=1M1σm2u˜m(k)×ωc+2πkN×expjωc+2πkNdmcx−xmcdm2+∑m=1M1σm2×u˜m*(k)×exp−jωc+2πkNdmc×∑m=1M1σm2×u˜m(k)×jωc+2πkN×expjωc+2πkNdmc×jωc+2πkN×x−xmcdm2+1cdm−(x−xm)2cdm3xmdm2∑m=1M2,

(A54) ∂2L∂x∂y=2AN2×Re∑k=−N/2N/2−1∑m=1M1σm2×u˜m*(k)×ωc+2πkNexp−jωc+2πkNdmcy−ymcdm∑m=1M×∑m=1M1σm2×u˜m(k)×ωc+2πkN×expjωc+2πkNdmcx−xmcdm∑m=1M+∑m=1M1σm2×u˜m*(k)×exp−jωc+2πkN×dmc×∑m=1M1σm2×u˜m(k)×jωc+2πkN×expjωc+2πkNdmc×x−xmcdm×y−ymcdm×jωc+2πkN−cdm∑m=1M.

The expressions for $\frac{\partial^{2}L}{\partial x\partial z}$, $\frac{\partial^{2}L}{\partial y^{2}}$, $\frac{\partial^{2}L}{\partial y\partial z}$, and $\frac{\partial^{2}L}{\partial z^{2}}$ are analogous. Note that the only random variables in the second derivatives of L are the frequency components ${\tilde{u}}_{m}\left(k\right)$. If the expectation operator is applied to the second derivatives, there will be terms of the form

(A55) $$\begin{array}{cc} E{\tilde{u}}_{m}^{*}\left(k\right){\tilde{u}}_{p}\left(k\right) & =E\left({\tilde{s}}_{m}^{*}\left(k\right)+{\tilde{w}}_{m}^{*}\left(k\right)\right)\left({\tilde{s}}_{p}\left(k\right)+{\tilde{w}}_{p}\left(k\right)\right)= \end{array}$$

(A56) $$\begin{array}{cc} \; & =E\left({\tilde{s}}_{m}^{*}\left(k\right){\tilde{s}}_{p}\left(k\right)\right) \end{array}$$

(A57) $$\begin{array}{cc} \; & =\exp\left(j\left(\omega_{c}+2\pi k/N\right)\left(d_{m}-d_{p}\right)/c\right)E{\left|\tilde{s}\left(k\right)\right|}^{2} \end{array}$$

(A58) $$\begin{array}{cc} \; & =N\sigma_{s}^{2}\exp\left(j\left(\omega_{c}+2\pi k/N\right)\left(d_{m}-d_{p}\right)/c\right),\;\mathrm{for}\;m\neq p, \end{array}$$

and

(A59) $$E{\tilde{u}}_{m}^{*}\left(k\right){\tilde{u}}_{m}\left(k\right)=N\sigma_{s}^{2}+N\sigma_{m}^{2}.$$

These equalities are used to derive the elements of the FIM. The derivation is somewhat tedious but straightforward and yields

(A60) $$\begin{array}{cc} I_{11} & =\beta \sum_{m=1}^{M}\sum_{p=1}^{M}\frac{1}{\sigma_{m}^{2}\sigma_{p}^{2}}\frac{x-x_{p}}{cd_{p}}\left(\frac{x-x_{p}}{cd_{p}}-\frac{x-x_{m}}{cd_{m}}\right), \end{array}$$

(A61) $$\begin{array}{cc} I_{12} & =\beta \sum_{m=1}^{M}\sum_{p=1}^{M}\frac{1}{\sigma_{m}^{2}\sigma_{p}^{2}}\frac{x-x_{p}}{cd_{p}}\left(\frac{y-y_{p}}{cd_{p}}-\frac{y-y_{m}}{cd_{m}}\right), \end{array}$$

(A62) $$\begin{array}{cc} I_{13} & =\beta \sum_{m=1}^{M}\sum_{p=1}^{M}\frac{1}{\sigma_{m}^{2}\sigma_{p}^{2}}\frac{x-x_{p}}{cd_{p}}\left(\frac{z-z_{p}}{cd_{p}}-\frac{z-z_{m}}{cd_{m}}\right), \end{array}$$

(A63) $$\begin{array}{cc} I_{22} & =\beta \sum_{m=1}^{M}\sum_{p=1}^{M}\frac{1}{\sigma_{m}^{2}\sigma_{p}^{2}}\frac{y-y_{p}}{cd_{p}}\left(\frac{y-y_{p}}{cd_{p}}-\frac{y-y_{m}}{cd_{m}}\right), \end{array}$$

(A64) $$\begin{array}{cc} I_{23} & =\beta \sum_{m=1}^{M}\sum_{p=1}^{M}\frac{1}{\sigma_{m}^{2}\sigma_{p}^{2}}\frac{y-y_{p}}{cd_{p}}\left(\frac{z-z_{p}}{cd_{p}}-\frac{z-z_{m}}{cd_{m}}\right), \end{array}$$

(A65) $$\begin{array}{cc} I_{33} & =\beta \sum_{m=1}^{M}\sum_{p=1}^{M}\frac{1}{\sigma_{m}^{2}\sigma_{p}^{2}}\frac{z-z_{p}}{cd_{p}}\left(\frac{z-z_{p}}{cd_{p}}-\frac{z-z_{m}}{cd_{m}}\right), \end{array}$$

where

(A66) $$\beta =\frac{2\sigma_{s}^{2}}{\frac{1}{\sigma_{s}^{2}}+\sum_{m=1}^{M}\frac{1}{\sigma_{m}^{2}}}\left(N\omega_{c}^{2}-2\pi \omega_{c}+\frac{N^{2}+2}{3N}\pi^{2}\right).$$

The CRBs are then given by

(A67) $$\begin{array}{cc} \mathrm{Var}\hat{x} & \geq {\mathrm{CRB}}_{x}={\left[I{\left(\alpha \right)}^{-1}\right]}_{11}, \end{array}$$

(A68) $$\begin{array}{cc} \mathrm{Var}\hat{y} & \geq {\mathrm{CRB}}_{y}={\left[I{\left(\alpha \right)}^{-1}\right]}_{22}, \end{array}$$

(A69) $$\begin{array}{cc} \mathrm{Var}\hat{z} & \geq {\mathrm{CRB}}_{z}={\left[I{\left(\alpha \right)}^{-1}\right]}_{33}. \end{array}$$

The bound on the mean squared error of the transmitter’s location estimate is then

(A70) $$\begin{array}{cc} \mathrm{CRB} & ={\mathrm{CRB}}_{x}+{\mathrm{CRB}}_{y}+{\mathrm{CRB}}_{z}\\ \; & =\frac{K_{11}+K_{22}+K_{33}}{\det I\left(\alpha \right)}\\ \; & =\frac{I_{22}I_{33}-I_{23}^{2}+I_{11}I_{33}-I_{13}^{2}+I_{11}I_{22}-I_{12}^{2}}{\det I\left(\alpha \right)}, \end{array}$$

where $K_{ij}$ is the cofactor of the $\left(i,j\right)$th element of $I\left(\alpha \right)$. If the transmitter’s *z*-coordinate is known (the 2D localization case), the last row and column of $I\left(\alpha \right)$ are removed to obtain the FIM for the 2D case,

(A71) $$I^{\left(2D\right)}=\;\left[\begin{array}{cc} I_{11} & I_{12}\\ I_{12} & I_{22} \end{array}\right].$$

So, the CRB for 2D localization is

(A72) $$\begin{array}{cc} {\mathrm{CRB}}^{\left(2D\right)}= & {\mathrm{CRB}}_{x}^{\left(2D\right)}+{\mathrm{CRB}}_{y}^{\left(2D\right)} \end{array}$$

(A73) $$\begin{array}{cc} = & \frac{I_{11}+I_{22}}{I_{11}I_{22}-I_{12}^{2}}. \end{array}$$

Under similar assumptions as in the known sequence scenario, starting from the derived expressions, and with the approximations

(A74) $$\begin{array}{cc} \; & N\omega_{c}^{2}-2\pi \omega_{c}+\frac{N^{2}+2}{3N}\pi^{2}\approx N\omega_{c}^{2}, \end{array}$$

(A75) $$\begin{array}{cc} \; & 1+\sum_{m=1}^{M}{\mathrm{SNR}}_{m}\approx 1,\;\;\;\mathrm{for}\;\mathrm{low}\;\mathrm{SNRs}, \end{array}$$

(A76) $$\begin{array}{cc} \;\; & 1+\sum_{m=1}^{M}{\mathrm{SNR}}_{m}\approx \sum_{m=1}^{M}{\mathrm{SNR}}_{m},\;\;\;\mathrm{for}\;\mathrm{high}\;\mathrm{SNRs}, \end{array}$$

it can be shown that the CRB approximately depends on ${\mathrm{SNR}}_{0}$, the carrier frequency, and the number of samples as follows:

(A77 $$\mathrm{CRB}∼1/{\mathrm{SNR}}_{0}^{2},\;\mathrm{low}\;\mathrm{SNRs},$$

(A78) $$\mathrm{CRB}∼1/{\mathrm{SNR}}_{0},\;\mathrm{high}\;\mathrm{SNRs},$$

(A79) $$\mathrm{CRB}∼1/\omega_{c}^{2},$$

(A80) CRB∼1/N.
